# Graduate Teaching Facilities in London Hospitals

**Published:** 1906-08-11

**Authors:** 


### Graduate Teaching Facilities in London Hospitals.

### NATIONAL HOSPITAL FOR PARALYSIS, QUEEN SQUARE, BLOOMSBURY.

### DETERMINATION OF THE ACTION OF MUSCLES.

This important centre of graduate teaching in London has been well supported by the members of the medical profession during the last session. The following notes of a demonstration will indicate the manner in which early anatomical knowledge is brought back to assist in the work of diagnosing nervous diseases and how important a knowledge of the enervation and accurate action of muscles is in such cases.

### Landouzy's Paralysis.

This is allied to pseudo-hypertrophic paralysis, and illustrates how a correct knowledge of the action of muscles is actually necessary for working out cases of muscular palsy and muscular atrophy. In this case the eyes, though shut, could be easily opened, showing paralysis of the levator palpebra muscles. If the patient tried to whistle the mouth assumed a chink-like form, and the lower lip was overhanging. He could show the teeth, showing he had the levator anguli oris; he had lost the zygomatics. He had the elevators of the upper lip. He had also marked paralysis and deformity of the muscles of the shoulders. Muscles of the thumb were active—quite different from a case of progressive muscular atrophy.

Nothing was wrong with the forearm until the supinator longus (brachio radialis) was tested by making the patient flex the elbow. It was practically gone—a characteristic of this condition. The triceps was somewhat larger than the biceps. A rough way of testing its strength was by using the little, middle, and forefingers respectively. Both scapulæ were deformed, the base sloping towards the spine on both sides, due to the absence of the trapezius and of the serratus magnus.

*Anatomical Note.*—The serratus magnus draws the base of the scapula forward as in pushing, and the lower fibres acting on the lower angle rotate the bone so as to elevate the point of the shoulder. It steadies the scapula and allows the deltoid to elevate the humerus, and keeps the lower angle of the scapula in contact with the chest wall.

The action of the serratus is seen by putting the arm forward. Patient has the deltoid, and can raise the arm as far as the horizontal line, but no farther. The further movement is carried on by the serratus.

*Anatomical Note.*—The acromial portion of the deltoid abducts the arm to the position of a right angle with the trunk. The clavicular portion draws it forwards and the spinal portion backwards. The origin of the muscle corresponds with the insertion of the trapezius. The upper fibres of the trapezius elevate the outer end of clavicle and the point of the shoulder. The middle fibres approximate the scapula to the spine. The entire muscle draws the scapula to the spine as in shrugging the shoulders. Acting from their insertion they extend the head and incline the neck towards the same side, the face being directed to the opposite.

An important use of this muscle is to steady the scapula, this being necessary before the deltoid can elevate the humerus. In this case the scapula projects backwards more and more as soon as the arm is brought forward. The loss is also indicated by pushing the arm back and making a movement as in swimming. If the trapezius is present it would then be seen rising as far down as the twelfth dorsal vertebræ from the spinous processes. The levator anguli scapulæ originates from the first four cervical transverse processes.

The latissimus dorsi is acting fairly well.

*Anatomical Note.*—The arm being raised, this muscle draws it downwards and backwards, producing at the same time internal rotation, as when the hands are crossed behind the back.

The pectoral muscles are also deficient. This is found by making the patient carry the arm inwards.

The pectoralis major muscle is divisible into two portions—clavicular and sternocostal. The patient has the upper fibres and a small piece of the lower, which can be demonstrated by making him press very hard. On lifting the arm up in the air the upper fibres of the pectoralis major are brought out. Acting both together the arm is carried inwards.

Thus the pectoral part and the sternal part have diametrically opposite action. Acting together they carry the arm inwards horizontally.

*Anatomical Note.*—Acting from its origin the pectoralis major adducts the arm, draws the arm forward—*i.e.*, flexes it at shoulder joint by its clavicular portion and rotates it inwards.

The whole point of this case is that there is no affection of the spinal cord or of the peripheral nerves. The disease is in the muscles themselves—allied to pseudo-hypertrophic paralysis. This class of case is named after Landouzy, of the Hospital Laënnec, who first described it and established that there was no affection of the cord, and that it was a disease of the muscles themselves, and not of the central nervous system. In disease of the cord the grouping of the muscles is different. In this case all muscles below the elbow, with the exception of the supinator longus (brachio-radialis), remain unaffected.

### A Case of Weakness of Movement of the Muscles of the Hand.

There are three ways of ascertaining the actions of a muscle: (1) Anatomical method; (2) Faradising a muscle (Duchesne's); and (3) the method which, I believe, is the only right one, the physiological method—the examination of the action of the muscle on the living body. The fallacy about the first two methods is that although movements on the dead body are produced, one cannot say whether the muscle takes part in the same movements during life.

The left hand is perfectly well, but the right hand, when closing the fingers, goes round. The patient can extend the fingers and thumb very well, but cannot extend the wrist owing to weakness of the extensors. At the same time she flexes the fingers she flexes the wrist, and the hand tends to go inwards.

She has all the movements of the interossei, a little weakness of the small muscles of the hand. She has got her legs spastic; anæsthesia of the arm and trouble with the sphincters. Probably it is a case of myelitis, affecting the arms and the legs.

To answer the question How it comes that the patient is able to extend the fingers but unable to extend the wrist, one has to examine the muscles and to see how they can be divided in groups.

In the list of muscles you have (1) prime movers, those which do the principal work—produce the action required—thus flexors produce the movement of flexion; (2) synergic muscles; (3) fixation muscles; (4) antagonistic muscles. The synergics are not antagonistic, although they are often confused with them. The antagonistics to the flexors are the extensors. The synergics are not the extensors of the fingers, but the extensors of the wrist; and, to explain further, all muscles which pass over more than one joint are synergic—*e.g.* the muscles which flex the fingers pass over the wrist and metacarpal joints and the next joints to be inserted into the base of the middle phalanx.

If mere flexion of the fingers and not of the wrist is required, other muscles must prevent the flexion of the wrist—namely, the extensors of the wrist (antagonistics). This can easily be proved by placing the finger on the tendon of the extensor carpi radialis. It is felt contracting whenever it is strongly grasped and the fingers are closed up. The synergic muscles are those which prevent a muscle from acting on joints which are not required to act. The interesting point in this case is that the wrist cannot be kept out of action whenever the fingers are flexed. In this case, whenever the woman tried to grasp, the hand went round inwards because the flexors of the fingers not only flex the fingers, but also the wrist. There was nothing to prevent the hand going round because the patient had paralysis of the extensors of the wrist.

Sometimes one gets paralysis of the interossei with claw hand, and inability to flex the metacarpo-phalyngeal joint, or to extend the last two phalyngeal joints. Paralysis of the interossei takes away the power of flexing that joint, and the hand remains in a state of hyperextension of the metacarpo-phalyngeal joint.

### A Case Showing Paralysis of Both Trapezii.

Four years ago the patient had involuntary twitching of the eyes, and of the head to the right and of both arms. To relieve this the spinal accessory nerve was divided. The patient shows the scapula slightly deformed. In cases of complete absence of trapezius there is some deformity of scapula rather similar to what is got in paralysis of the serratus magnus, but differing in this particular—in cases of deformity of the serratus magnus the deformity begins at the commencement of the action, and gets more and more apparent until the arm gets horizontal; whereas in paralysis of the trapezius the deformity comes on at first, and then disappears by the time the arm is horizontal. Thirdly, in cases of paralysis of the serratus magnus the patient is unable to raise the arm beyond the horizontal line; but in paralysis of the trapezius he can carry the arm up to the highest point without much difficulty because he has the serratus unimpaired. The deformity is much less, and in paralysis of the trapezius one can see the serrations of the serratus, whereas in paralysis of the serratus they are absent. Another way of showing trapezius deformity is to push the arm back, and in this case there is no appearance of the trapezius. The upper fibres of the trapezius in this case are absent on one side, but present on the other.

The levator anguli scapulæ is apparent.

### Tabes, with Wasting of both Forearms.

It is very unusual in tabes (locomotor ataxy) to have any wasting of the limbs. This case had a specific history 22 years ago. Seven years ago wasting commenced with progressive general emaciation, and two years ago the patient became unable to walk. The attack has lasted about two years. He has got the signs of tabes—fixed pupils (Argyle Robinson's pupils), but the unusual thing in the condition is the wasting of the forearm: notice the extreme wasting of the extensors. The interossei are very much wasted, and also the muscles of the ball of the thumb. Patient is unable to separate his fingers and his thumb; the first interossei is absent. He has the deep flexors and the deep extensors of the thumb. With that exception he has lost all the small muscles of the thumb; all he can do is to flex and extend the thumb. He has no power to adduct or abduct or oppose the thumb to the fingers. The thumb faces the same way as the fingers—called the Simian thumb—and due to the fact that the movements of opposition and rotation of the thumb are lost. He has got the movements of the wrist fairly well. The other symptoms are fixed pupils, no knee-jerks; he has got co-ordination. He has also sharp pains in the right foot, all signs of tabes, accompanied by a great atrophy of muscles of the hand. Sometimes in tabes you get atrophy of the muscles of the leg, but it is very rare indeed to get so much atrophy of the hand.

### Paralysis of the Deltoid and Supra and Infra Spinatus.

The patient is aged 18, and some time ago had severe pains following on influenza. After two weeks he noticed that the arm was painful for two or three days, and that he had sharp pains running down the hands and arms.

The point is that he has lost the power of the deltoid on the right side. He cannot move the arm away from the side. The way to test the movements of the deltoid is to abduct the arm and ask the patient to try and hold the arm up. The other muscles affected in this case are the supra and infra spinatus. He is also a little weak in the depressors of the arm. The triceps is slightly weak. The biceps quite strong. The diagnosis in this case lies between a case of myelitis and neuritis affecting the circumflex, supra-scapular, and also the musculo-spiral slightly.

All these cases shown indicate the movements of the hand and the shoulder-joint, and especially the movements of the serratus magnus and also of the trapezius. Another movement which brings out the presence or absence of the serratus magnus best is the movement of lunging. You can tell at once from the amount of strength the patient puts in whether it is present or not.

